# Intra-subject test-retest reliability for auditory-evoked functional near-infrared spectroscopy responses: effects of systemic physiology correction

**DOI:** 10.1117/1.NPh.12.1.015015

**Published:** 2025-03-17

**Authors:** Victoria C. Sinfield, Dalton Aaker, Abigail Metzger, Yunjie Tong, Maureen J. Shader

**Affiliations:** a Purdue University, Weldon School of Biomedical Engineering, West Lafayette, Indiana, United States; b Purdue University, Department of Speech, Language, and Hearing Sciences, West Lafayette, Indiana, United States

**Keywords:** systemic physiology augmented functional near-infrared spectroscopy, test-retest reliability, systemic physiology, hemodynamic signal denoising, intra-subject variability

## Abstract

**Significance:**

Functional near-infrared spectroscopy (fNIRS) is a valuable neuroimaging tool for non-invasively measuring hemodynamic changes in response to neural activity, particularly in auditory research. Although fNIRS shows strong test-retest reliability at the group level, individual-subject level reliability is often compromised by systemic noise.

**Aim:**

We investigate how correcting for systemic-physiological signals affects reliability in single-subject fNIRS data.

**Approach:**

fNIRS data were collected from one participant over 10 sessions during a passive auditory task. Using general linear modeling, six correction approaches were compared: no correction, physiology correction, short-channel correction, short-channel + physiology correction, short-channel + physiology + lag correction, and short-channel + tCCA correction.

**Results:**

Intraclass correlation coefficient analysis revealed that physiology correction yielded the highest test-retest reliability score, whereas short-channel correction had the lowest. These results align with previous findings suggesting that global systemic artifacts bolster reliability, and regressing such artifacts enhances the clarity of the observed neuronal response, as supported by visual comparisons of raw and denoised signals.

**Conclusions:**

We highlight the impact of correcting for extra-cerebral signals in single-subject auditory research and demonstrate that, while incorporating short channels in fNIRS data collection may reduce reliability, it offers a more accurate representation of the neuronal response.

## 1 Introduction

Functional near-infrared spectroscopy (fNIRS) is a non-invasive neuroimaging tool for measuring hemodynamic changes associated with task-evoked neural activity. It relies on the foundational principle of neuro-vascular coupling—a link between neural activity and the delivery of oxygen via a complex vascular mechanism.^1^^,^^2^ Specifically, fNIRS measures changes in the concentration of oxygenated and deoxygenated hemoglobin in cortical blood, exploiting the absorption and scattering characteristics of near-infrared light when passing through biological tissue.^3^ Since its development for research applications in humans in the 1970s, fNIRS has been used in a variety of research contexts in both adults and children.^4^

Notably, fNIRS is particularly useful in auditory research for several reasons.^5^ Unlike functional magnetic resonance imaging (fMRI)—similarly, a hemodynamic-based neuroimaging method that measures changes in the concentration of oxygenated and deoxygenated hemoglobin in cortical blood, also known as the blood oxygenation level-dependent signal—fNIRS is virtually silent, eliminating potential sound interference on auditory stimuli. Although fMRI is physically confining and restrictive, requiring a high level of participant compliance to achieve clear images, fNIRS measurements are less susceptible to movement artifact, allowing participants to sit upright in a more naturalistic listening position. Near-infrared light signals used in fNIRS measurements are not susceptible to electromagnetic interference, allowing participants to safely use listening devices such as hearing aids and cochlear implants. As such, fNIRS offers a favorable imaging modality in a variety of contexts within the field of auditory neuroscience.

A major caveat to the aforementioned benefits is that while fNIRS measurements are largely reliable and repeatable at the group level, relatively poor test-retest reliability is reported at the single-subject level.^6^^–^^8^ Although group-level designs are commonly used to address many research questions, they often fail to prioritize individual-level measurement reliability.^9^ As a result, within-subject reliability becomes a critical component when studying clinical populations or evaluating the effectiveness of a treatment or intervention. Factors limiting the within-subject test-retest reliability of fNIRS measurements include differences in instrumentation,^10^ lack of spatial information for accurate cortical localization of the optodes,^11^ and contamination from non-neural, extra-cerebral, or systemic-physiological signals in the data.^12^^,^^13^ Scholkmann et al.^14^ classified six signal components present in every recorded fNIRS signal according to the (i) source (intra- versus extra-cerebral), (ii) task (evoked versus non-evoked), and (iii) cause (neuronal versus systemic). The primary signal of interest for fNIRS experiments is the one component characterized as intra-cerebral, task-evoked, and neuronal in origin. Isolating the signal of interest from other extra-cerebral and systemic components can be challenging given that the hemodynamic changes induced by fluctuations in systemic physiology (e.g., increasing/decreasing heart rate or respiration rate) are larger than the hemodynamic changes evoked by the brain itself.^15^ Group-level analysis of the fNIRS signal of interest from multiple participants overcomes some of these limitations, leading to higher degrees of test-retest reliability.^16^^,^^17^ However, such challenges are more difficult to mitigate at the single-subject level.

A new approach, systemic physiology augmented fNIRS (SPA-fNIRS), offers a promising method for improving the within-subject test-retest reliability of fNIRS measurements.^18^ SPA-fNIRS accounts for confounding physiology components in fNIRS data by measuring brain activity while simultaneously measuring fluctuations in systemic physiology signals. This approach recognizes the complex interplay between the body and the brain, allowing for more precise isolation of the neural signal of interest from other signal components. Short-separation channels, which measure signals originating from superficial, extra-cerebral tissues, are commonly used to remove noise from fNIRS data. Wyser et al.^19^ demonstrated that short-channel regression alone can enhance test-retest reliability at the single-subject level. SPA-fNIRS builds on this foundation by not only incorporating short-channel regression but also integrating systemic physiology signals, which account for stimulus-evoked changes in cardio-respiratory and autonomic activity that can accompany stimulus-evoked changes in neural activity.^14^ Resting-state functional connectivity metrics have also been shown to correlate with oscillations in extra-cerebral tissue oxygenation.^20^ SPA-fNIRS enables the quantification of multiple systemic-physiology signals that can be categorized as nuisance regressors in the data analysis, increasing the likelihood of accurate data interpretation, and potentially, improving the reliability of the results.^14^

Improving the reliability and signal quality of fNIRS data is particularly critical in auditory research contexts. Despite the many advantages of fNIRS as an imaging modality for auditory experiments, its sensitivity for estimating auditory-evoked brain activity in deep neural structures may be limited. Unlike fMRI, which allows for imaging of the entire brain, optical signals transported through the scalp and skull have a limited penetration depth into the surface of the cortex of ∼2  cm in adults.^21^ As a result, measurable changes in oxygen concentration from deeper brain structures, such as the primary auditory cortex, often suffer from small effect sizes and a higher susceptibility to disruptions from systemic physiology and other nuisance variables.^22^ The inherent limitation of optical neuroimaging signals combined with the anatomical depth of auditory brain structures poses an additional challenge to the reliability of fNIRS measures in auditory research.

The aim of this study was to investigate the test-retest reliability for auditory-evoked fNIRS measurements collected over 10 separate recording sessions from a single participant with and without extra-cerebral and systemic-physiology correction using SPA-fNIRS. A standard block-design paradigm was used to measure passive auditory-evoked brain activity in response to speech within relevant auditory- and speech-processing regions of interest. The experiment was repeated twice a day over five consecutive days for a total of 10 recording sessions from the same listener.

## 2 Materials and Methods

### 2.1 Ethical Considerations

This study was approved by the Institutional Review Board of Purdue University, in accordance with internationally accepted ethical guidelines for research involving human subjects. Informed consent was obtained from the participants prior to the experiment, and all procedures adhered to the ethical standards of the institutional oversight body.

### 2.2 Stimuli

Auditory speech stimuli consisted of 20 trials of two to three concatenated sentences taken from the English AzBio speech-in-noise test.^23^ Stimuli were presented at random while a visual fixation cross was shown on the screen in front of the participant. Each stimulus trial was between 5.18 and 7.31 s in duration with an average duration of 6.1 s. The auditory stimuli were presented at 60 dBA.

### 2.3 Participants

The participant was a 23-year-old male with normal audiometric hearing in both ears at octave frequencies between 250 and 8000 Hz. The participant was fluent in American English and reported no known history of hearing, cardiovascular, or neurological issues. The participant abstained from caffeinated beverages for 1 week prior and for the duration of the 5-day experiment.

### 2.4 Procedure

The participant was seated 1 m directly in front of a monitor screen in a sound-attenuating booth. A fixation cross was presented on the monitor throughout the duration of the experiment. Stimuli were presented through ER-3C insert earphones bilaterally. A block-design protocol was used in which 20 trials of auditory stimuli, averaging 6.1 s in duration as outlined in Sec. 2.2, were randomly presented throughout the experiment followed by an inter-stimulus interval between 15 and 30 s before the initiation of the next trial. In addition, 20 trials of a control condition consisting of 6 s of silence were presented at random followed by the same 15 to 30 s inter-stimulus interval. The experiment was passive in nature; the participant was instructed to sit still and quietly to listen to the sentences. The total testing time was ∼20  min. The identical procedure was repeated twice a day for five consecutive days. The first recording each day was taken at ∼9:00 A.M. and the second at ∼3:00 P.M.

### 2.5 fNIRS Acquisition

**Fig. 1:**
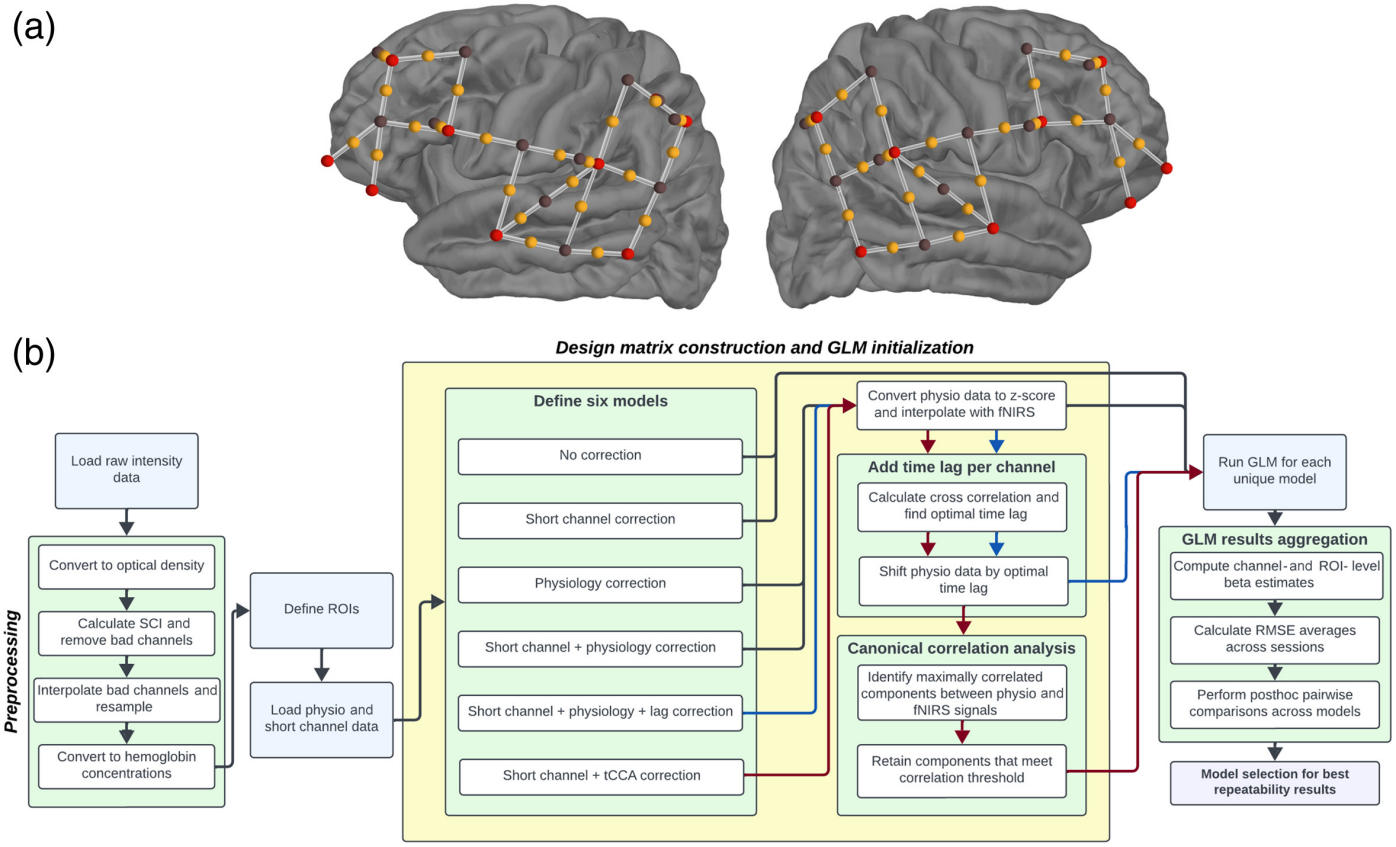
(a) Montage containing the location of all sources and detectors. Optodes were placed over the left and right inferior frontal gyrus and the left and right superior temporal gyrus (including the primary and secondary auditory cortices). Source locations are shown in red and detector locations are shown in black. Channels are shown as white lines with an orange dot representing the midpoint. (b) Flowchart outlining the algorithm pipeline from data collection to statistical analyses. The yellow box, titled “Design matrix construction and GLM initialization,” highlights the primary focus of this work.

Data were acquired using a NIRx NIRSport2 continuous wave fNIRS device and a NIRx WINGS module for physiology measurements (NIRSport2, NIRx Medizintechnik GmbH, Berlin, Germany). The data were originally sampled at 5.1 Hz and subsequently down-sampled to 0.6 Hz for analysis. During preprocessing, the signal was converted from optical density to oxygenated hemoglobin and deoxygenated hemoglobin concentrations using the Beer-Lambert Law with a partial pathlength factor of 0.1. A high-pass finite impulse response filter with a cutoff frequency of 1.35 Hz and a transition bandwidth of 0.1 Hz was used during the calculation of the scalp coupling index to identify and remove poorly coupled channels. The NIRSport2 device used 16 light-emitting diode (LED) sources (two near-infrared light illuminators with wavelengths of 760 and 850 nm) and 14 detectors. Eight short-channel detectors were included in the montage to collect extra-cerebral activity. The montage [shown in Fig. 1(a)] designated 38 source-detector pairs placed ∼3  cm apart. Sources were placed at locations AF7, F7, F3, FC5, T7, CP5, P7, P3, AF8, F8, F4, FC6, T8, CP6, P8, and P4 with short-channel detectors placed on F3, FC5, CP5, P3, F4, FC6, CP6, and P4. Detectors were placed on locations F5, FC3, C5, TTP7h, TP7, CP3, P5, F6, FC4, C6, TTP8h, TP8, CP4, and P6. The montage *a priori* regions of interest were selected according to the Jülich atlas provided by the fOLD toolbox^24^^,^^25^ to include the left and right primary and secondary auditory cortices and the left and right inferior frontal gyri. The correspondence between the regions of interest and the 10-10 locations is detailed in Table S1 in the Supplementary Material.

### 2.6 Systemic-Physiology Acquisition

Three primary systemic-physiological processes influence fNIRS measurements: (1) the cardiorespiratory system (heart rate, respiration rate, and oxygen saturation),^26^ (2) the state of the autonomic nervous system (body temperature and galvanic skin response (GSR)),^27^^,^^28^ and (3) body movement.^29^ The NIRx WINGS device was used to measure multiple physiological signals of interest at a sampling frequency of 500 Hz. These data were later downsampled and interpolated to match the sampling rate of the fNIRS signals. Natural fluctuations in the participant’s cardiorespiratory system were measured via a heart rate monitor and pulse oximeter (${\mathrm{SpO}}_{2}$) on the earlobe and a bioimpedance-based respiration monitor on the chest, which detects changes in electrical impedance caused by variations in lung air volume and chest expansion during breathing. Respiratory sinus arrhythmia is one of the major underlying mechanisms regulating the cardiorespiratory system and refers to the coupling between the respiratory and cardiac systems in which heart rate increases during inhalation and decreases during exhalation. The coupling and synchronization between these systems allow for efficient pulmonary gas exchange at the level of the lungs.^30^^,^^31^ The rate of respiration depends on the surrounding environment and stimuli present. When a stimulus that demands oxygen consumption is present, the sensory input system signals the brain to increase respiration rate, increasing the amount of oxygen being inhaled and carbon dioxide being exhaled.^32^ The autonomic nervous system was measured via a temperature probe on the palm and a skin conductance monitor secured to the other palm. The skin conductance monitor measures the GSR, which refers to a rapid change in the electrical resistance of the skin in response to a stimulus. As part of the arousal mechanism in the autonomic nervous system, the GSR is controlled through the sympathetic cholinergic nerve supply to the skin or the sweat glands. An increase in arousal resulting from an external stimulus will cause an increase in sweat gland activity, increasing the conductance through the skin, which is measurable with the sensor on the palm.^33^ Each physiology sensor collected real-time data during the fNIRS recording. Our analysis used these time-series signals to account for variance in the fNIRS data originating from non-neural systemic sources. For the purposes of this study, the NIRx WINGS device was used to measure six peripheral physiological signals: photoplethysmography (PPG), heart rate, oxygen saturation (${\mathrm{SpO}}_{2}$), respiration, body temperature, and GSR.

### 2.7 Data Analysis

Analysis was accomplished using MNE,^34^^,^^35^ Nilearn,^36^ and MNE-NIRS.^37^ Data preprocessing and block-averaging were performed to visualize the morphology of the hemodynamic response following each stimulus condition, followed by a quantitative analysis to evaluate the influence of systemic physiology (nuisance signals) on the within-subject detection of auditory-evoked brain activity.

### 2.8 Block averaging

Response waveforms were calculated using a standard analysis pipeline summarized in the “Preprocessing” section of Fig. 1(b). Raw intensity signals were converted to optical densities, and channels with a scalp coupling index (SCI)<0.7—alluding to insufficient optode-to-scalp connection—were removed from the analysis.^38^ Motion artifacts were removed with temporal derivative distribution repair (TDDR),^39^ and short-separation channel data were subtracted to filter out non-cortical blood oxygenation influences.^40^ Both TDDR and short-channel subtraction were performed exclusively for the block averaging analysis and were not implemented on the data that was subsequently fed into the general linear model (GLM). The signal was then converted to oxyhemoglobin (HbO) and deoxyhemoglobin (HbR) estimates using the modified Beer-Lambert Law^41^ and bandpass filtered (0.02 to 0.4 Hz) to remove slow drifts in the signal. Epochs corresponding to 1 s before stimulus onset and 15 s after stimulus onset were extracted for the speech stimulus and control trials to best capture the hemodynamic response of each stimulus type.

### 2.9 General Linear Modeling

For quantitative analysis, the raw fNIRS responses were fit to a GLM of the canonical SPM hemodynamic response model,^42^ convolved with a boxcar function of 3 s in width. Modeling of the hemodynamic response function followed the best-fit parameters for passive auditory fNIRS experiments reported by Luke et al.^37^ Prior to the GLM analysis, no filtering was performed on the raw hemodynamic signal, aligning with insights from Huppert,^43^ which suggests that filtering the signal may distort the statistical properties of noise and degrade the analysis by removing or altering the temporal correlations inherent in both the neural signal and physiological noise, potentially leading to biased estimates and reduced GLM accuracy. No pre-whitening or pre-coloring steps were applied in the GLM analysis. Short channels underwent the same preprocessing as long channels, and neither included filtering prior to performing the GLM. MNE-Python^44^ was employed to run the regression analysis, which uses an ordinary least squares GLM. The GLM analysis provided estimates (beta values) for each stimulus condition at each channel location for each of the 10 sessions. These beta values act as regression coefficients that represent the strength or weight of a particular regressor (i.e., the stimulus condition) on the response variable (i.e., the hemodynamic response). To better estimate neural responses while accounting for potential extra-cerebral, systemic factors, six GLM models were constructed: a No correction model (control), a Physio correction model (including systemic physiology signals—heart rate, respiration, ${\mathrm{SpO}}_{2}$, GSR, temperature, and PPG—as regressors), a short-separation channel (SS) correction model (with only short channels as regressors), a SS + Physio correction model, a SS + Physio + Lag correction model, and a SS + tCCA correction model. The Physio, SS + Physio, and SS + Physio + Lag correction models included systemic physiological signals as regressors. In these models, all physiological signals were converted to z-scores, down-sampled, and interpolated with the fNIRS signal to align the time series. Prior to this, the GSR signal was low-pass filtered with a cutoff frequency of 5 Hz to remove high-frequency noise—no other physiological signals went through additional filtering. The SS + Physio + Lag correction model incorporated a temporal lag in the GLM framework to account for transit time between the peripheral physiology signals and the fNIRS signal.^19^^,^^20^ This was achieved by allowing a time shift range of 0 to 30 s for each regressor and then determining the optimal time lag for each source-detector channel. The optimal lag was identified as the one yielding the highest cross-correlation value between the regressor and the individual channel, as implemented by Abdalmalak et al.^45^ in their group-level work. The average optimal time lags for each physiological signal were as follows: heart rate, 5.50 s; respiration, 28.17 s; ${\mathrm{SpO}}_{2}$, 18.83 s; PPG, 0.67 s; GSR, 20.83 s; and temperature, 10.00 s. The SS + tCCA correction model is built on the time-lagged approach by applying temporally embedded canonical correlation analysis (tCCA), mirroring the method developed by von Lühmann et al.^22^ In this model, canonical correlation analysis (CCA) was performed between the time-lagged physiological signals and the fNIRS signal, identifying latent components that maximized the shared variance between the two datasets. Only components meeting the pre-determined threshold of $\rho_{\text{thresh}}=0.3$ were included as regressors in the GLM. In addition, all physiological signals in this model were low-pass filtered with a cutoff frequency of 0.5 Hz prior to the CCA, consistent with von Lühmann’s approach. Each of these models generated beta values and root mean square error (RMSE) values across all sessions. The RMSE represents the root mean square of the error term in the GLM, quantifying the average magnitude of the prediction error between the observed and predicted fNIRS response. Region of interest (ROI) calculations were performed through the weighted averaging of beta values across channels within each region, using weights equal to the inverse of the standard error of the GLM fit for each channel.^46^^,^^47^

### 2.10 Denoising the Raw Signal

To visualize the individual effects of short-channel, systemic physiology, and time-lagged physiology correction on the hemodynamic response signal, we generated estimated time-series signals that represent the fNIRS response after regressing out these non-neuronal contributions. This was achieved using a GLM with the following matrix structure in Eq. (1): 
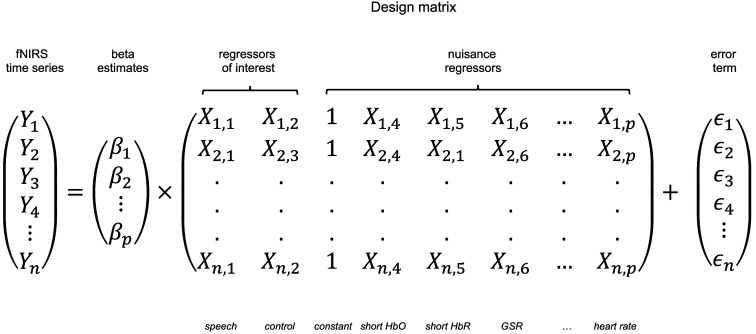
(1)

By subtracting each regressor’s weighted contribution from the raw fNIRS signal, based on this matrix structure, we reduce the dimensionality of noise and can demonstrate the effects of each correction model on the signal.

We derived five denoised signals to examine the impact of different correction models on the fNIRS time series. The general equation for the denoised signal is given by

$$Y_{\text{filtered}}=Y_{\text{raw}}-\beta X$$ (2)

where $Y_{\text{filtered}}$ represents the denoised signal, $Y_{\text{raw}}$ is the original fNIRS time series (shortened to $Y_{\text{raw}}$ for simplicity), and βX represents the weighted contribution of the specific nuisance regressors (depending on the correction model) subtracted from the hemoglobin time series. The regressors X correspond to systemic physiological signals, short-channel signals, or their combinations, and β represents the corresponding estimated regression coefficients. In all the correction models, the beta values were computed as part of the GLM where the contributions of each regressor were estimated simultaneously. Each correction model operated by subtracting a specific set of regressors, weighted by their respective regressor coefficient, as described below:

#### 2.10.1 Systemic physiological effects regressed out

In this model, we subtract the effects of systemic physiological signals. The denoised signal is given by

$$Y_{\text{filtered}}=Y_{\text{raw}}-\underset{i}{\sum }\beta_{\text{physio},i}X_{\text{physio},i}$$ (3)

where $\beta_{\text{physio},i}$ represents the regression coefficient for each individual systemic physiological effect and $X_{\text{physio}},i$ is the corresponding matrix of physiological data for each signal collected in this study (i.e., heart rate, respiration, GSR, temperature, ${\mathrm{SpO}}_{2}$, and PPG).

#### 2.10.2 Short-channel effects regressed out

In this model, we regress out only the short-channel data and the denoised signal is calculated as

$$Y_{\text{filtered}}=Y_{\text{raw}}-\underset{j}{\sum }\beta_{SS,j}X_{SS,j}$$ (4)

where $\beta_{SS,j}$ is the regression coefficient for each of the short-channel effects (one for each chroma—HbO and HbR) and $X_{\text{physio},j}$ is the corresponding matrix of the short-channel signal.

#### 2.10.3 Both physiological and short-channel effects regressed out

In this combined model, we account for both systemic physiological and short-channel effects simultaneously. The denoised signal is given by

$$Y_{\text{filtered}}=Y_{\text{raw}}-\underset{i}{\sum }\beta_{\text{physio},i}X_{\text{physio},i}-\underset{j}{\sum }\beta_{SS,j}X_{SS,j}$$ (5)

#### 2.10.4 Time-lagged physiological effects and short-channel effects regressed out

To refine the correction further, this model subtracts the time-lagged physiological effects, along with the short-channel effects. The denoised signal is calculated as

$$Y_{\text{filtered}}=Y_{\text{raw}}-\underset{i}{\sum }\beta_{\text{lag}\_\text{physio},i}X_{\text{lag}\_\text{physio},i}-\underset{j}{\sum }\beta_{SS,j}X_{SS,j}$$ (6)

where $\underset{i}{\sum }\beta_{\text{lag}\_\text{physio},i}$ represents the time-lagged physiological effects, accounting for temporal relationships between physiological signals and the fNIRS data.

#### 2.10.5 Latent components in physiology effects and short-channel effects regressed out

To refine the correction further, this model subtracts the effects of latent components derived from time-lagged physiological signals, along with the short-channel effects. The denoised signal is calculated as

$$Y_{\text{filtered}}=Y_{\text{raw}}-\underset{k}{\sum }\beta_{tCCA,k}X_{tCCA,k}-\underset{j}{\sum }\beta_{SS,j}X_{SS,j}$$ (7)

where $\sum_{k}\beta_{tCAA,k}$ represents the effects of the latent components extracted from the physiological signals, as derived through tCCA.

It is important to note that there is no parallel filtered signal for the No correction model, as this would involve subtracting no regressor effects, leaving the original time series unchanged. By regressing systemic noise, we effectively minimize its influence on the hemodynamic response signal. Consequently, we get closer to observing the neuronal contribution to the fNIRS signal associated with the auditory-evoked task.

### 2.11 Test-retest Reliability

Test-retest reliability of the hemodynamic response amplitude estimates across sessions was assessed by using the intraclass correlation coefficient (ICC) for each correction model across all ROIs. In accordance with the recommendation of Li et al.^48^ for calculating ICC in fNIRS studies, we selected ICC(3,k), as the experimental design is most accurately represented by a two-way mixed effects model where raters are random and targets are fixed; our primary interest lies in the consistency of the estimates averaged across raters for each model. The ICC calculations were conducted using the Python statistical package *pingouin*, with sessions serving as the targets, ROIs as the raters, and beta amplitude estimates as the ratings. The beta values were retrieved from ROI-level analyses and filtered to solely include the speech condition. The ICC was interpreted based on the following criteria: poor (<0.40), fair (0.40 to 0.59), good (0.60 to 0.74), and excellent (0.75 to 1.00) reliability.^48^

## 3 Results

### 3.1 Block-Averaging Results

**Fig. 2:**
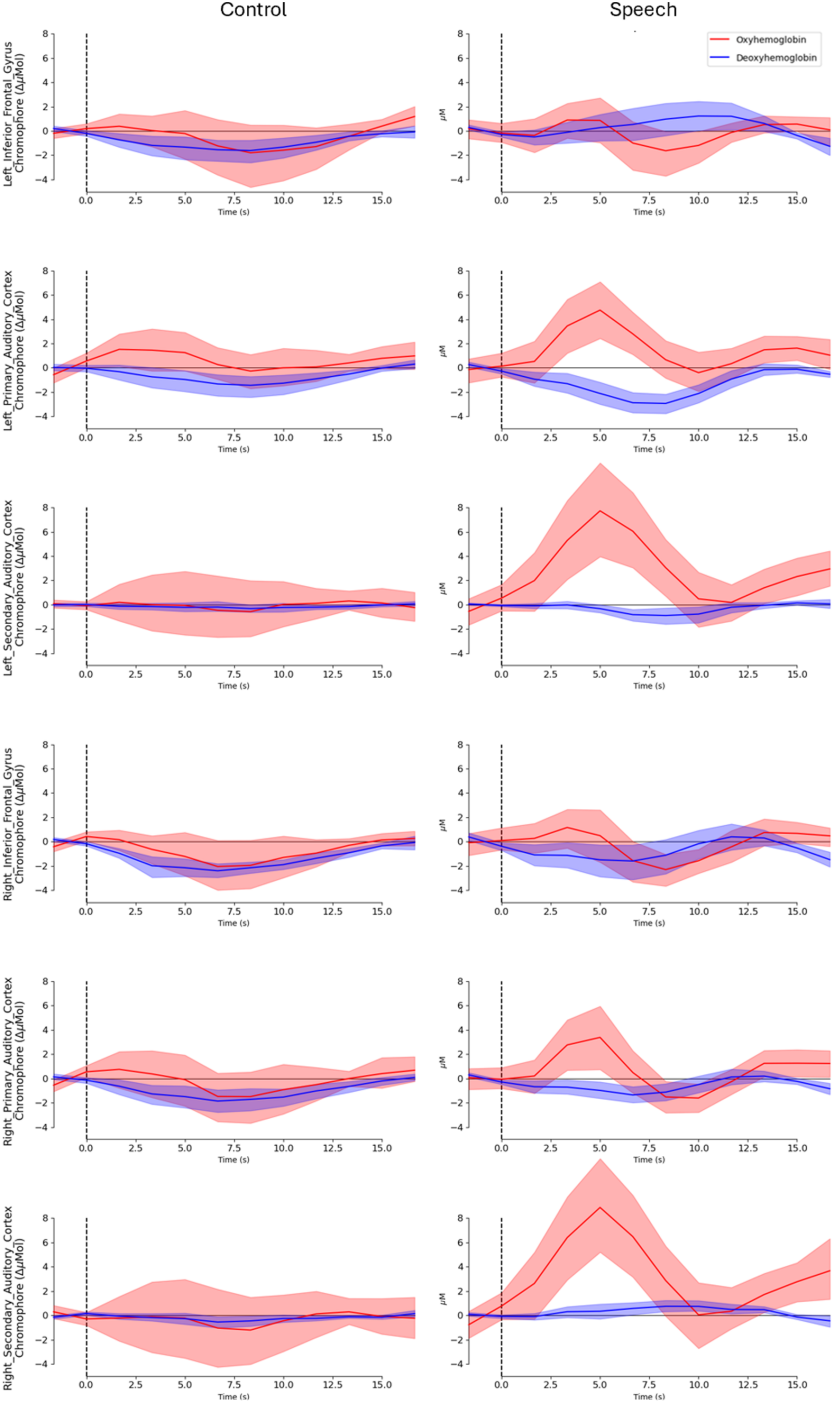
Block-averaged waveforms from each ROI for the control (silence) stimulus and the auditory speech stimulus across all 10 recording sessions from the same listener. Each individual recording contains 20 control trials and 20 stimulus trials, resulting in a total of 200 trials per condition. Shaded areas represent 95% confidence intervals.

Block-averaged waveforms representing the mean hemodynamic response within each ROI across all 10 recording sessions are shown in Fig. 2 for both control and speech events. The morphology of the waveforms reflected typical hemodynamic response characteristics of an auditory-evoked passive task. Auditory-evoked responses were isolated to the left and right auditory regions of interest. Thus, these regions were highlighted moving forward into the GLM analysis.

**Fig. 3:**
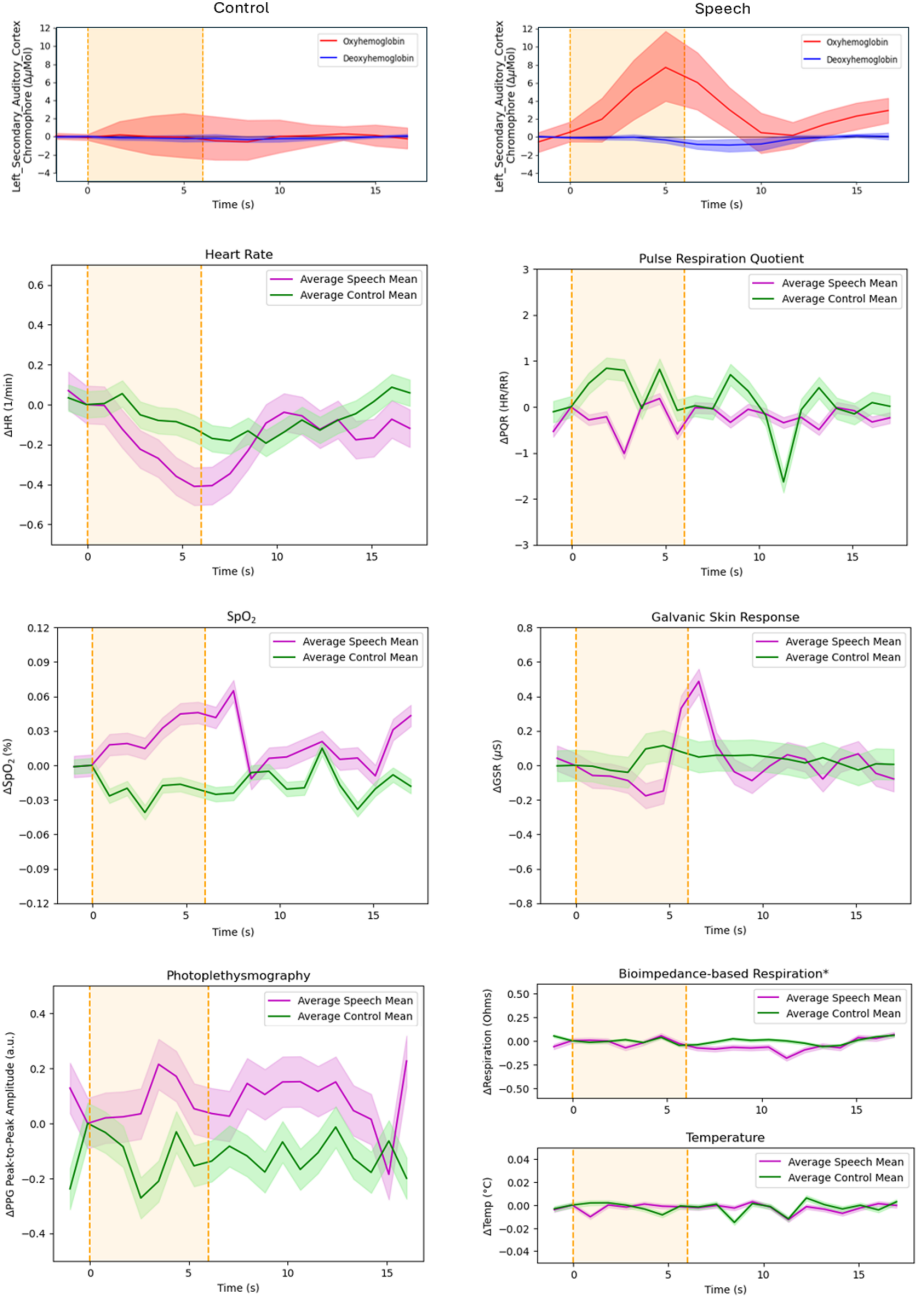
Block-averaged physiological data for heart rate, PRQ, ${\mathrm{SpO}}_{2}$, GSR, respiration, and temperature aligned with block-averaged speech and control responses in the left secondary auditory cortex ROI across all 10 recording sessions from the same listener. Shaded areas represent 95% confidence intervals. Physiology block averages were obtained using the same processing pipeline as the hemoglobin response waveforms in Fig. 2. However, raw physiological signals were not filtered prior to block averaging as the hemodynamic signals were. *The respiration block average omits session 7 due to poor attachment of the respiration belt.

To assess the relationship between task-evoked hemodynamic responses and systemic physiology, Fig. 3 presents block averages of the collected physiological signals, as well as a calculated pulse-respiration quotient (PRQ) (heart rate/respiration rate),^49^ alongside block-averaged hemodynamic responses in the left secondary auditory cortex for both speech and control events. During speech events, on average, heart rate tends to decrease, respiration stays constant due to the relationship between stimulus length (6 s) and typical human respiration rate (12 to 20 BrPM),^50^ and temperature also stays relatively constant. ${\mathrm{SpO}}_{2}$ and GSR both increase following speech trials with a slight delay from the onset of the event. PPG shows an increase in peak-to-peak amplitude for the speech condition and the PRQ shows a slight decrease, correlating with the observed change in heart rate. Block-averaged waveforms of the hemodynamic response for speech events of each individual session are illustrated in Figure S1 of the Supplementary Material.

### 3.2 GLM Results

**Fig. 4:**
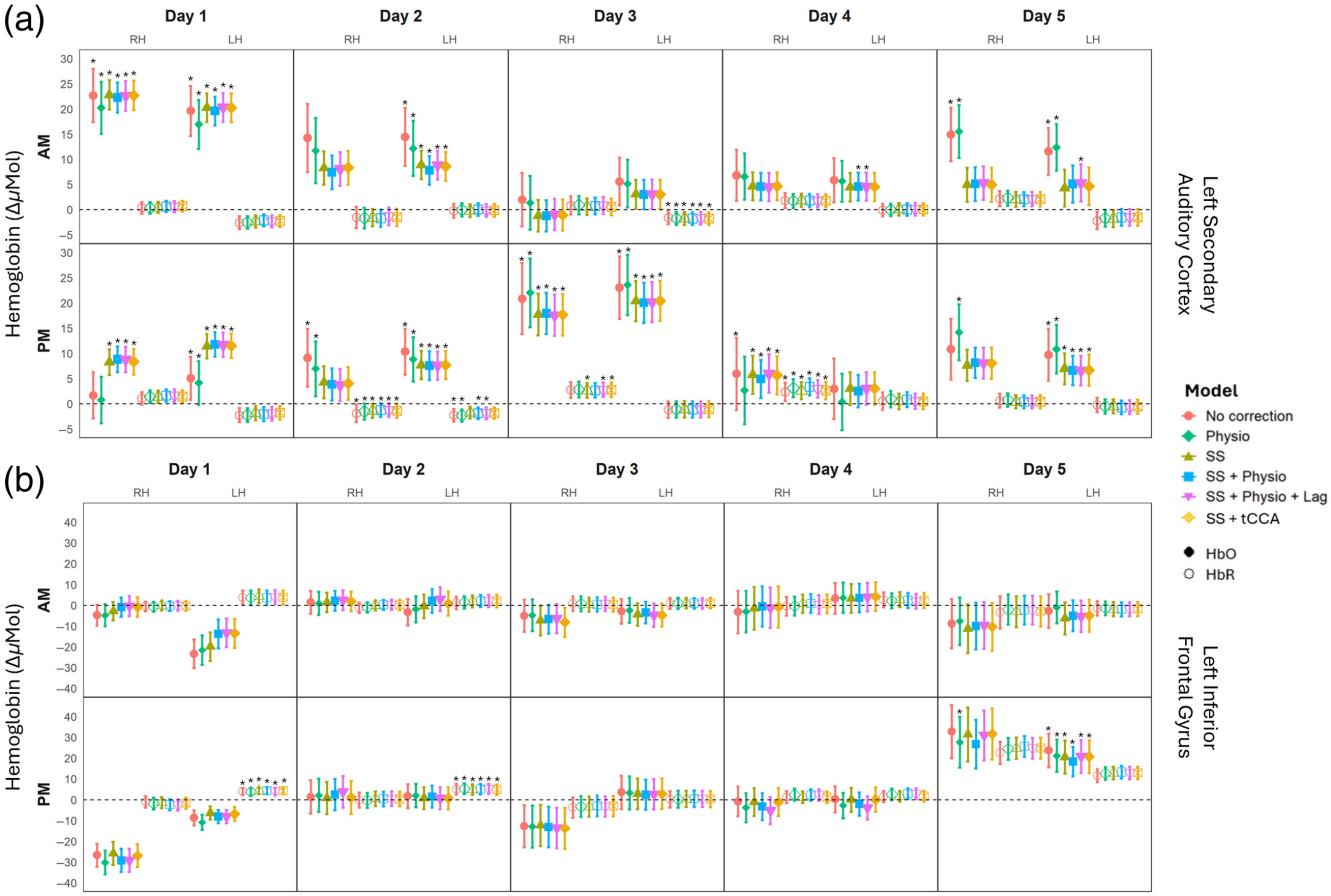
GLM beta estimates for auditory-evoked responses – both HbO (filled) and HbR (open) – from (a) the left (LH) and right (RH) auditory, and (b) the left (LH) and right (RH) inferior frontal gyrus ROIs for six model types: no correction, short-channel correction, physiology correction, combined short-channel and physiology correction, combined short-channel, physiology, and time lag correction, and combined short-channel and tCCA-derived physiological latent component correction. Each panel represents the results for each day of testing. The top row of each plot displays results from each morning session (AM); the bottom row displays results from each afternoon session (PM). Stars above the plotted betas indicate that the beta estimate is statistically significantly different from zero, using multiple one-sample t-tests where Bonferroni correction was applied to the alpha level (α=0.0025). The Bonferroni correction method, which was applied to account for multiple comparisons, is highly conservative, and as such, reduces the likelihood of Type I errors. However, it can increase the risk of Type II errors, particularly in studies with smaller sample sizes, as in this case. Thus, some analyses may fail to detect significant effects, requiring cautious interpretation in an effort to minimize false positives while maintaining sensitivity to true effects.

HbO and HbR beta values for each session were extracted from each GLM model—those from both hemispheres of the secondary auditory cortex and the inferior frontal gyrus are highlighted in Fig. 4 with the latter serving as a secondary control given the lack of auditory-evoked response in that region. Plotting of these betas and their respective RMSE values as error bars indicates that HbO beta values are much more susceptible to change when established using different correction models for both regions, whereas HbR values are minimally susceptible, if at all. Notably, for the secondary auditory cortex, the No correction and Physio correction models produce similar results, whereas the other four models exhibit comparable patterns. Conversely, for the inferior frontal gyrus, there is very little change across all correction models. In addition, no consistent trends are observed concerning the time of day. Interestingly, when larger HbO response amplitudes are present, they tend to be consistent across hemispheres during single recordings. However, this consistency does not persist throughout that same day, as response amplitudes can vary significantly, with higher levels in the morning and lower levels in the afternoon, or vice versa.

**Fig. 5:**
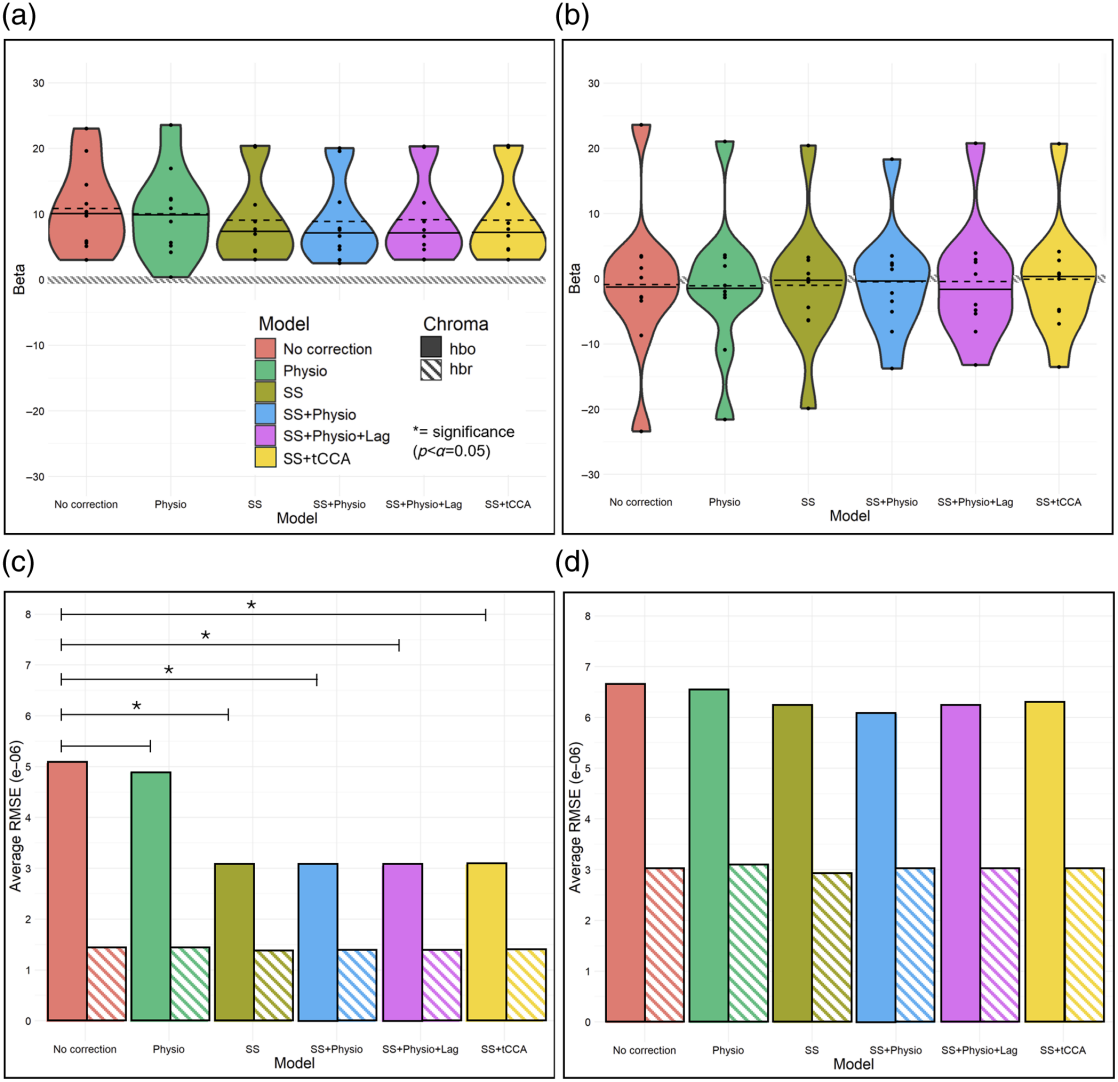
(a, c) Violin plot of (a) left secondary auditory cortex and (c) left inferior frontal gyrus beta values from all 10 sessions across all six models with dashed and solid horizontal lines representing mean and median, respectively, and the black dots representing the actual beta value data points. The shaded line marks when the beta is zero. (b, d) Average RMSE values of (b) left secondary auditory cortex and (d) left inferior frontal gyrus betas across all 10 sessions for HbO and HbR in the left secondary auditory cortex for all six correction models. Average RMSE values for SS correction, SS + Physio correction, SS + Physio + Lag, and SS + tCCA correction are all statistically significantly less than No correction solely for HbO. No correction and Physio correction models are not significantly different from one another in either HbO or HbR. *Post hoc* pairwise comparisons were performed to draw these conclusions.

The violin plots in Fig. 5 display the distribution of HbO beta values across all 10 sessions in (a) the left secondary auditory cortex and (b) the left inferior frontal gyrus for all six correction models. A larger distribution of beta values across the 10 sessions suggests more variance in the results and infers poorer test-retest reliability in that specific ROI. Within the left secondary auditory cortex, the SS, SS + Physio, SS + Physio + Lag, and SS + tCCA correction models all have a similar range of distribution, as well as similar means and medians. This suggests that these four models have comparable consistency in beta value estimates across multiple sessions. The SS, SS + Physio, SS + Physio + Lag, and SS + tCCA models have lower variances compared with the No correction and Physio models, which implies that the latter two models have poorer consistency in the beta estimates across multiple sessions. Specifically, the No correction and Physio models have variances of 43.05 and 45.91, respectively, whereas the SS, SS + Physio, SS + Physio + Lag, and SS + tCCA models have variances of 42.40, 40.32, 41.24, and 42.20, respectively. Within the inferior frontal gyrus, all models have similar means and medians—centering near zero (as expected for a region with no task-evoked response)—and all models have high variance (averaging 99.39 across models), suggesting a diminished effect of correction models on the beta estimates in this region.

The bar graphs in Fig. 5 illustrate the average RMSE values across all 10 sessions for both HbO and HbR beta estimates specific to (c) the left secondary auditory cortex and (d) the left inferior frontal gyrus. For left secondary auditory cortex HbO values, the SS, SS + Physio, SS + Physio + Lag, and SS + tCCA correction models have much lower average RMSE values than the No correction and Physio models. This difference was determined to be statistically significant based on results derived from *post hoc* pairwise comparisons using estimated marginal means. No statistically significant difference was found between the models’ RMSE values for the inferior frontal gyrus. Pairwise comparisons also revealed no significant difference between any of the models’ average RMSE values for HbR for either region. This aligns with previous observations based on Fig. 4. Although Fig. 5(c) displays results specific to the left secondary auditory cortex ROI, the same trend in average RMSE values among models is observed for the other regions of interest in which significant auditory-evoked activity was present. Thus, these results indicate that the SS, SS + Physio, SS + Physio + Lag, and SS + tCCA correction models allow for significantly improved (i.e., decreased) error when compared with the No correction and the Physio correction models.

### 3.3 Individualizing the Effects of Systemic Physiology and Denoising the Raw Signal

**Fig. 6:**
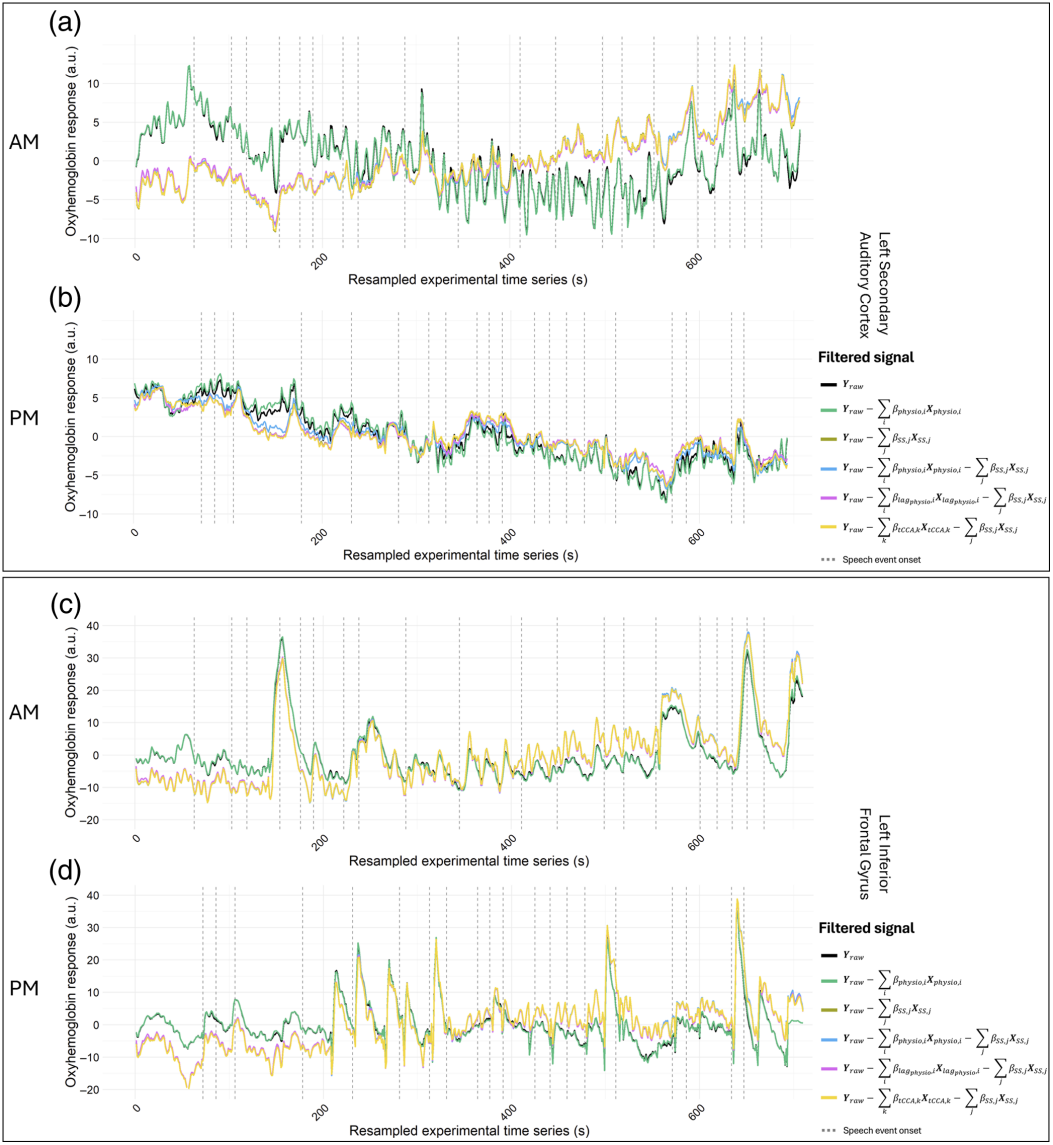
Individual channel time series of the full experiment when regressing out extra-cerebral and systemic physiology noise from the raw signal, compared to the original, raw fNIRS time series, for both an (a, c) AM and (b, d) PM session. Panels (a) and (b) correspond to the channel chosen between source 8 and detector 6 over the left secondary auditory cortex; panels (c) and (d) correspond to the channel chosen between source 1 and detector 1 over the left inferior frontal gyrus (an ROI without an auditory-evoked response). Labels of each signal correspond to the equations signifying the regressors being subtracted from the raw fNIRS response (i.e., “$Y_{\text{raw}}-\underset{i}{\sum }\beta_{\text{physio},i}X_{\text{physio},i}$” signifies that physiology components were subtracted [regressed out] from the raw signal).

With the goal of further identifying the effects of extra-cerebral and systemic physiology noise, Fig. 6 presents the raw oxyhemoglobin response time series at an individual channel level for one morning session and one afternoon session in both a region with (left secondary auditory cortex) and without (left inferior frontal gyrus) a significant auditory-evoked response, alongside mathematically derived versions of this signal in which nuisance regressors (e.g., short channels, physiology, and lagged physiology) are regressed out of that signal [Eq. (2)]. In both regions, the $Y_{\text{raw}}-\underset{i}{\sum }\beta_{\text{physio},i}X_{\text{physio},i}$ synthesized signal relates most closely to the original oxyhemoglobin raw signal, being nearly identical. Regressing out short channels ($Y_{\text{raw}}-\underset{j}{\sum }\beta_{SS,j}X_{SS,j}$) leads to a significant alteration in the response signal’s structure, characterized by greater smoothing and a reduction in the magnitude of fluctuations compared to the raw signal. Similar effects are observed when short channels and physiology are both regressed out, as well as when lag and canonical correlation are considered.

### 3.4 Test-Retest Reliability Metric

The ICC, which serves as an objective single-value metric used to quantify the test-retest reliability across sessions and all ROIs, was calculated for each model for both HbO and HbR: No correction (HbO: 0.720, HbR: 0.773), Physio correction (HbO: 0.785, HbR: 0.767), SS correction (HbO: 0.640, HbR: 0.782), SS + Physio correction (HbO: 0.654, HbR: 0.770), SS + Physio + Lag correction (HbO: 0.683, HbR: 0.772), and SS + tCCA correction (HbO: 0.664, HbR: 0.778). These results, also outlined in Table 1, indicate that the Physio correction model yielded the highest test-retest reliability rating and falls in the category of excellent ICC (0.75 to 1.00), whereas the other models’ ICC values are interpreted as good (0.60 to 0.74) for the HbO chroma. The ICC values for the HbR chroma were highly consistent across all correction models.

**Table 1:** ICC values for each correction model across all ROIs for both HbO and HbR.

Model	HbO ICC	HbR ICC
No correction	0.720	0.773
Physio correction	0.785	0.767
SS correction	0.640	0.782
SS + Physio correction	0.654	0.770
SS + Physio + Lag correction	0.683	0.772
SS + tCCA correction	0.664	0.778

## 4 Discussion

The primary objective of this study was to explore and quantify the effects of extra-cerebral and systemic physiological factors on intra-subject test-retest reliability for auditory-evoked fNIRS measurements using SPA-fNIRS. Addressing these factors is crucial for improving the reliability and usability of fNIRS across a wide range of applications, as the technique’s sensitivity to physiological interference often complicates the interpretation of neural signals. This challenge is particularly critical in auditory research, where deep regions of interest (e.g., the auditory cortex) have inherently low signal-to-noise ratio due to greater susceptibility to interference from extra-cerebral and physiological sources. This makes it challenging to isolate true neural responses and negatively impacts measurement reproducibility. Effective correction is therefore essential to improve stability across repeated sessions and enhance fNIRS usability in auditory studies.

In this study, systemic physiology data were recorded simultaneously during the fNIRS recordings using the NIRxWINGS physiology module. The data were analyzed using standard GLM approaches with and without accounting for short channels and systemic physiology through six unique models: a No correction model (control), a Physio correction model (including systemic physiology signals such as HR, ${\mathrm{SpO}}_{2}$, and GSR as regressors), a SS correction model (introducing short channels as regressors),^51^ a SS + Physio correction model, a SS + Physio + Lag correction model (accounting for time delays between physiological and neural changes),^52^ and a SS + tCCA correction model (leveraging CCA to identify and regress out latent components shared between physiological and fNIRS signals). Test-retest reliability metrics quantified using the ICC metric were calculated for all analysis approaches to investigate the reliability of the results. Based on the theoretical framework of dimensionality reduction, it was hypothesized that accounting for extra-cerebral and systemic physiology signal components would improve the reliability of single-subject fNIRS recordings.^19^

The block-averaged waveforms in Fig. 2 revealed expected hemodynamic response characteristics for auditory stimuli within the auditory regions of interest, which provided confirmation of a successful experimental setup.^53^ The trends in physiological signals illustrated in Fig. 3 align with the hypothesis that participants tend to hold their breath and experience an autonomic system reaction at the onset of stimulus presentation, which aligns with past research findings.^13^^,^^33^ The observed delays in ${\mathrm{SpO}}_{2}$ and GSR increase can be attributed to differences in physiological response mechanisms. Although the heart rate responds quickly to autonomic nervous system signals, ${\mathrm{SpO}}_{2}$ increases more slowly due to the time required for oxygen transport and distribution in the bloodstream.^54^ Similarly, GSR, which reflects changes in skin conductance due to sweat gland activity, takes longer to manifest as the sympathetic nervous system triggers sweat secretion gradually.^55^ Physiological signals did not vary significantly during control (silence) events, which mirrors the behavior of the hemodynamic response. Generally, the trends in physiological signals align with previous findings from Scholkmann et al.,^14^ with slight variation likely deriving from a difference in stimulus type (i.e., auditory versus visual). Such trends lay the groundwork for further exploration of the relationship between extra-cerebral signals and hemodynamic responses through GLM analyses.

The results of these analyses, conducted on six unique models (i.e., No correction, Physio correction, SS correction, SS + Physio correction, SS + Physio + Lag correction, SS + tCCA correction) and summarized in Figs. 4 and 5, provide an improved understanding of the effects of physiological signals, short channels, time lag, and CCA on fNIRS readings. These figures also highlight the comparison between a region with a significant auditory-evoked response (secondary auditory cortex) and one serving as a secondary control without such a response (inferior frontal gyrus), offering insights into the regional differences in these effects. A crucial initial distinction in these results lies in the pronounced difference between HbO and HbR beta estimates. Although HbO estimates and their errors are model-dependent, those for HbR are consistent across different models with consistently low errors. This finding aligns with past group-level studies, indicating that HbO signals are more vulnerable to physiological interference.^28^^,^^45^ This susceptibility may be generally attributed to oxygenated blood’s dominance in the bloodstream over deoxygenated blood. In addition, prior research has suggested that these differences may arise from distinct responses of arterioles and venules during autonomic system activity.^56^^,^^57^

With a focus on the HbO estimates, an important insight from this work is that all models perform equally well in producing beta estimates that are statistically significant in regions with expected task-evoked activity. However, in terms of the actual accuracy of the estimates themselves, differences across models become evident. The consideration of physiological signal regressors alone does not significantly alter the beta estimates of speech events or their associated errors when compared with no correction. Instead, when coupled with short-channel regressors, lag, and CCA, there is decreased error in the resulting beta estimates. This trend is further supported by post hoc RMSE analyses, which indicate that the models incorporating short-channel regressors, lag, and CCA consistently show reduced error in auditory regions with significant task-evoked activity. In regions with no significant task-evoked activity, such as the inferior frontal gyrus, consideration of physiological signal regressors does not significantly alter the beta estimates. This is expected given that in these regions, the beta estimates are no longer an estimate of the task-evoked response. Because there is no significant task-related signal, the correction models have minimal effect on the beta values in this area.

When expanding the analysis to assess reliability across all ROIs, as seen in the model-specific ICC calculations (Table 1), the following values resulted: No correction (HbO: 0.720, HbR: 0.773), Physio correction (HbO: 0.785, HbR: 0.767), SS correction (HbO: 0.640, HbR: 0.782), SS + Physio correction (HbO: 0.654, HbR: 0.770), SS + Physio + Lag correction (HbO: 0.683, HbR: 0.772), and SS + tCCA correction (HbO: 0.664, HbR: 0.778). It is essential to note that these ICC metrics are intended to measure the consistency of the neural signal across sessions in response to different correction methods rather than the consistency of the participant’s physiological or emotional states. In considering physiological signals (i.e., by including them as regressors), we aim to account for broader systemic across-session differences—such as variations in mood or circadian rhythm^58^—ensuring that the ICCs reflect the stability of the neural signals and not potentially confounding factors.^29^^,^^45^^,^^59^

The HbO ICC values from this study align with previous fNIRS test-retest reliability research, such as that conducted by Dravida et al.,^16^ which also evaluated intra-participant signal consistency, albeit with two subjects rather than one. Similar to their findings, our results indicate that regressing out short channels actually leads to a lower test-retest reliability score (0.640) while regressing out physiology yields a higher ICC (0.786). One possible explanation for this result could be the nature of the signals targeted by short channels versus physiological regressors. Short channels capture more localized superficial artifacts—such as scalp blood flow—which are often highly consistent signals.^40^^,^^60^^,^^61^ This consistency can inflate test-retest reliability by masking the true variability of the neural signal. When these extra-cerebral artifacts are removed through short-channel regression (as in the SS correction model), the observed reliability drops, potentially revealing the lower inherent stability of the neural signal. In contrast, physiological signals are typically measured peripherally and reflect broader systemic fluctuations that affect the brain in a more uniform way. Regressing out these peripheral signals could clean the data without drastically altering the more consistent aspects of the measured signal, preserving high test-retest reliability. This interpretation aligns with that of Dravida et al.,^16^ who noted that global systemic artifacts can bolster reliability, and regressing these artifacts from the raw signal can expose the true nature of the neural data. In contrast to HbO ICC values, the HbR ICC values showed negligible change and were consistent across all correction models. This corroborates with the previous discussion that HbR signals are less susceptible to physiological interference than HbO signals and thus are less likely to be affected by correcting for such noise.

These considerations help us address when short-separation channels might be the better choice despite lower ICC values. Although higher ICC values are generally preferred, as they suggest greater test-retest stability, a high ICC is not always desirable if it primarily reflects consistency in non-neuronal components of the signal. It is likely that using data that accounts for the removal of extra-cerebral and systemic noise provides a more valid measure of the neuronal signal’s consistency, free from the inflation caused by unaccounted noise. Therefore, although accounting for short channels, physiology, lag, and CCA may reduce the ICC, their inclusion is advantageous as it theoretically yields a more accurate representation of the neuronal response. In the absence of physiological measurement regression, the ICC could misleadingly reflect systemic noise rather than the neuronal response, inflating the apparent reliability of the data. That being said, short-separation channels, which are increasingly employed and available in fNIRS research, can account for much of this noise and should be employed to obtain the best estimation of the neural response, especially when physiological measurements are not available.

To further explore these insights, we sought to examine more closely how extra-cerebral and physiological factors influence the overall characteristics of the oxyhemoglobin response. Figure 6 displays how the fNIRS time series response would look if the effects of these factors were subtracted (regressed out) from the original raw signal in both the left secondary auditory cortex (which has a significant task-evoked response) and the left inferior frontal gyrus (which lacks a significant task-evoked response). This analysis revealed that short channels have the most pronounced effect on altering the response signal, leading to an overall reduction in signal fluctuation magnitude, whereas physiological regressors and lagging do not contribute as significantly. Short-channel signals primarily comprise contributions from physiological fluctuations in oxygenated and deoxygenated hemoglobin, as well as extra-cerebral influences such as scalp blood flow and tissue scattering, rather than from deeper cerebral activity. These components introduce rapid fluctuations into the fNIRS signal oftentimes larger than the brain-evoked hemodynamic fluctuations themselves.^15^^,^^40^ Thus, their regression effectively reduces these fluctuations, explaining the observed effects on the signal. Notably, this analysis reveals that when examining the entire time series, the correction models have similar effects on the signal in both regions. This is likely because the correction models are now being applied to the signals independent of the task-evoked responses. The visual evidence gathered from Fig. 6 aligns with the earlier ICC findings, further illustrating the substantial influence of short-channel correction on signal variability.

This work brings us closer to visualizing the primary signal of interest: the intra-cerebral, task-evoked signal of neuronal origin, by mitigating the influence of extra-cerebral and systemic noise. By incorporating models that account for these factors, we significantly reduce the impact of non-neuronal fluctuations on the fNIRS signal. These models, especially those that use time-lagged physiological signals and CCA, enhance the clarity of the neuronal response, even if they slightly reduce ICC values. Although a reduction in ICC may occur, the inclusion of these regressors, particularly CCA, also helps prevent overfitting by addressing collinearity among physiological signals. This is particularly important in situations in which multiple correlated physiological signals could otherwise distort the analysis, such as in active tasks. Though the approach outlined herein does not entirely isolate the neuronal signal—due to potential contributions from other unmeasured sources of noise—it significantly reduces the influence of non-neuronal factors, thereby enhancing the clarity of the observed neuronal response. We recommend that future researchers test multiple models to determine the most appropriate approach for their specific research context and task type, taking these considerations into account to optimize the accuracy and reliability of their results.

## 5 Limitations and Future Work

Although this study provides valuable insights into the effects of short channels and physiological signals on fNIRS data, several limitations should be acknowledged. First, the experimental task was passive, thus providing potentially weaker activation signals and greater susceptibility to extra-cerebral noise.^62^ In addition, the collection of physiology was limited to six physiological signals. Supplementary equipment to gauge other metrics could allow for the consideration of other potentially consequential auxiliary signals such as end-tidal ${\mathrm{CO}}_{2}$, which is known to reflect changes in cerebral vasculature and blood flow.^63^ Furthermore, although we accounted for a one-time lag per individual channel in our analysis, the body’s physiological processes are inherently dynamic, with varying delays across systems and time points.^52^ Thus, future work could benefit from exploring multiple lags to better capture the temporal complexity of these interactions. It must also be noted that this study was performed with solely one subject. Although it is hoped that this work provides a baseline from which to evaluate any single-subject test-retest reliability, future studies may require a larger pool of participants for the results to be more confidently translatable and generalizable. Moreover, future work should incorporate resting-state acquisitions to estimate physiological transfer functions prior to running the GLM,^64^^,^^65^ to establish a gold standard for evaluating ICC, beta estimates, and RMSE measurements. Finally, repeating the study with different fNIRS montages and different tasks—such as motor, cognitive, and visual paradigms—would allow for a systematic assessment of test-retest reliability across brain regions and tasks.

## 6 Conclusion

These findings have significant implications for the field of auditory neuroscience. By evaluating the impact of extra-cerebral and systemic noise on the test-retest reliability of single-subject fNIRS data, we highlight the importance of accounting for non-neuronal components to achieve more accurate measurements. In denoising the raw signal of extra-cerebral and systemic physiology noise by means of SPA-fNIRS, we effectively enhance the interpretability of fNIRS responses, particularly in the identification of relationships and potential correlations between auditory stimuli and brain activity. Specifically, our work demonstrates the crucial role of short-separation channels in explaining response signal variation, emphasizing the importance of their inclusion in future fNIRS acquisition setups, particularly within the context of single-subject testing. Although the regression of short channels, physiology, lag, and tCCA-derived latent components may reduce test-retest reliability, our systematic comparison of models suggests that a full model (i.e., SS + tCCA correction) may be optimal, as it consistently reduces root mean square prediction error, lowers the chance of overfitting, and ultimately provides a more accurate representation of the underlying neuronal signal, rather than one influenced by unaccounted noise. Overall, this work provides a clearer and more quantifiable understanding of the effects of short channels and systemic physiology on fNIRS data and establishes a methodological framework for improving intra-subject test-retest performance in auditory research.

## Supplementary Material

10.1117/1.NPh.12.1.015015.s01

## Data Availability

Code used for GLM analyses is publicly available on GitHub at the following URL: https://github.com/vsinfield22/SPA-fNIRS_test-retest_reliability. All data supporting the findings of this paper are publicly available in an Open Science Framework (OSF) repository titled “Intra-subject test-retest reliability for auditory-evoked fNIRS responses: Effects of systemic physiology correction,” at DOI https://doi.org/10.17605/OSF.IO/YJT6G.
