# Supplementary material for: Intra-subject test-retest reliability for auditory-evoked functional near-infrared spectroscopy responses: effects of systemic physiology correction
Source: Neurophotonics. 2025 Mar 17;12(1):015015. doi: 10.1117/1.NPh.12.1.015015 (PMC11924667; doi:10.1117/1.NPh.12.1.015015)
Supplement: Supplementary file 1 [file NPh_012_015015_SD001.pdf]

Supplementary Material

Region of Interest	10-10 Locations
Left primary auditory cortex	T7-C5, T7-TP7, CP5-TP7, CP5-CP3
Right primary auditory cortex	T8-C6, T8-TP8, CP6-TP8, CP6-CP4
Left secondary auditory cortex	CP5-P5, P3-CP3, P3-P5
Right secondary auditory cortex	CP6-P6, P4-CP4, P4-P6
Left inferior frontal gyrus	AF7-F5, F7-F5, F3-F5, FC5-F5, FC5-FC3, F3-FC3
Right inferior frontal gyrus	AF8-F6, F4-F6, F8-F6, F4-FC4, FC6-F6, FC6-FC4

Table S1. Correspondence between regions of interest and the 10-10 locations of the montage using the Jülich montage

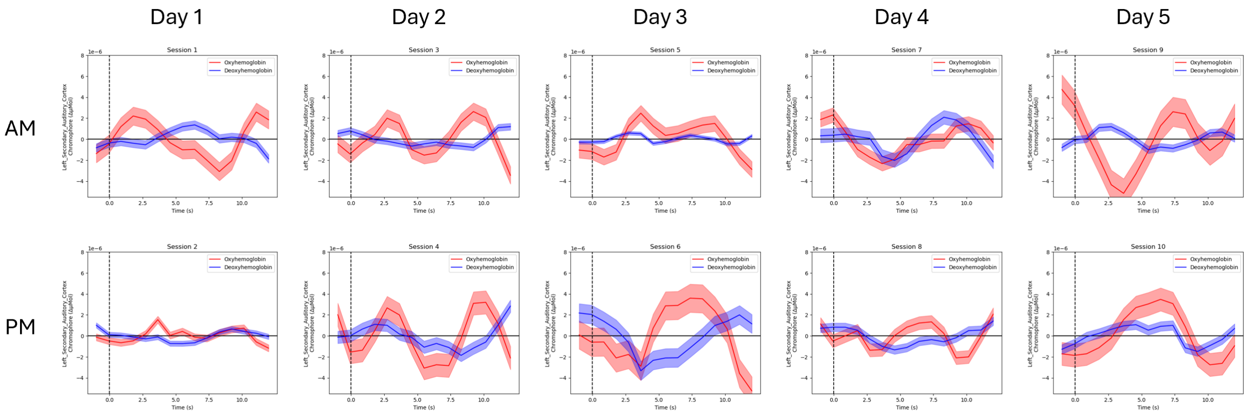

Figure S1. Session-specific block-averages of the fNIRS speech response for both HbO and HbR for all 10 sessions.
